# Identification and validation of a prognostic signature of autophagy, apoptosis and pyroptosis-related genes for head and neck squamous cell carcinoma: to imply therapeutic choices of HPV negative patients

**DOI:** 10.3389/fimmu.2022.1100417

**Published:** 2023-01-10

**Authors:** Zhaodi Nan, Yu Dou, Anwei Chen, Ketao Wang, Jintang Sun, Zhen Meng, Markus Neckenig, Dan Ai, Shaohua Liu, Zuoqing Dong, Chao Ma, Yufeng Cheng, Xun Qu

**Affiliations:** ^1^ Laboratory of Basic Medical Sciences, Qilu Hospital, Cheeloo College of Medicine, Shandong University, Jinan, China; ^2^ School and Hospital of Stomatology, Cheeloo College of Medicine, Shandong University, Jinan, China; ^3^ Shandong Key Laboratory of Oral Tissue Regeneration, Shandong University, Jinan, China; ^4^ Shandong Engineering Laboratory for Dental Materials and Oral Tissue Regeneration, Shandong University, Jinan, China; ^5^ Shandong Provincial Clinical Research Center for Oral Diseases, Shandong University, Jinan, China; ^6^ Department of Oral and Maxillofacial Surgery, Qilu Hospital, Cheeloo College of Medicine, Shandong University, Jinan, China; ^7^ Precision Biomedical Laboratory, Liaocheng People’s Hospital, Medical College of Liaocheng University, Liaocheng, China; ^8^ School of Pharmaceutical Sciences, Shandong University, Jinan, China; ^9^ Department of Radiation Oncology, Qilu Hospital, Cheeloo College of Medicine, Shandong University, Jinan, China

**Keywords:** head and neck squamous cell carcinoma, human papillomavirus, autophagy, apoptosis, pyroptosis, tumor microenvironment, prognosis, radiotherapy

## Abstract

**Introduction:**

An effective tool is needed to predict the prognosis of head and neck squamous cell carcinoma (HNSCC). Human papillomavirus (HPV) positive HNSCC patients generally have a favorable survival and a promising responsiveness to radiotherapy, chemoradiotherapy and checkpoint blockades. However, HPV negative patients, the majority of HNSCC patients, have been largely overlooked. Cell death has been involved in the therapeutic resistance of cancers. To this end, we aimed to identify the association of autophagy, apoptosis and pyroptosis-related genes with the prognosis of HNSCC, and construct a prognostic signature to predict the prognosis for HNSCC, especially for HPV negative HNSCC.

**Methods:**

Autophagy and apoptosis-related genes were obtained from Gene Set Enrichment Analysis (GSEA) website, and pyroptosis-related genes were obtained from GSEA and Gene Ontology (GO) database. We established the cell death index (CDI) based on RNA sequencing (RNA-seq) data and clinicopathological information from The Cancer Genome Atlas (TCGA) dataset. The prognostic value of CDI was verified by Kaplan-Meier, receiver operating characteristic (ROC) and univariate and multivariate Cox regression analyses in TCGA dataset, and validated with the datasets from Gene Expression Omnibus (GEO) and Qilu Hospital of Shandong University. We further assessed the immune microenvironment of patients with high and low CDI scores. Moreover, the expression of the signature genes in HNSCC cell lines were explored.

**Results:**

We found that CDI was an independent prognostic indicator for overall survival (hazard ratio 3.80, 95% confidential interval: 2.70-5.40, *P* < 0.001). Furthermore, HNSCC patients with high CDI scores obtained increased overall survival post radiation indicating benefits from radiotherapy of this subgroup. On the other hand, HPV negative HNSCC patients with low CDI exhibited increased checkpoint gene expressions, an inflamed tumor microenvironment and an enriched immune response-related functions, suggesting the potential benefits from checkpoint immunotherapies of this subgroup. Moreover, we validated the baseline and induced expressions of above 16 genes in two HPV negative HNSCC cell lines, CAL27 and SCC-15.

**Discussion:**

We established a prognostic signature and emphasized its implements in the therapeutic choices of HPV negative HNSCC patients, the majority and the poor outcome population of HNSCC.

## Introduction

Head and neck cancer (HNC) accounts for over 870,000 new cases and 440,000 deaths worldwide annually (1). Over 90% of HNC is head and neck squamous cell carcinoma (HNSCC) (2). Surgical resection with or without adjuvant radiation or chemoradiation is the primary therapeutic option for HNSCC patients (3). However, studies showed that adjuvant therapies did not significantly improve overall survival, as compared with surgical resection alone (4, 5). The programmed death 1 (PD-1) immune checkpoint blockades have shown durable responses and survival improvements in the patients with recurrent or metastatic HNSCC (6, 7). Unfortunately, an estimated 85% of patients with recurrent or metastatic HNSCC are not responsive to this treatment (6, 7). Therefore, novel approaches to predict clinical outcomes and therapy responsiveness are needed for HNSCC patients, so as to stratify them for precise and multi-disciplinary therapy strategies.

The prevalence of human papillomavirus (HPV) related HNSCC is increasing, whereas, HNSCC related with tobacco and alcohol use is slowly declining (4, 8). HPV positive HNSCC is biologically distinct from HPV negative HNSCC (8). Moreover, HPV positivity has shown association with favorable survivals and promising responsiveness to radiotherapy, chemoradiotherapy and PD-1 blockades (3, 9, 10). However, the need, to stratify HPV negative patients, the majority of HNSCC patients and also the subgroup with poor prognosis, for optimized therapy strategies, has been largely overlooked. Therefore, the efficacy of the prognostic model for HPV negative HNSCC patients should be emphasized.

Radiation and chemotherapy modulate the tumor microenvironment (TME) initiated by cell death (11, 12). Moreover, emerging evidences have revealed that tumor cell death associates with therapy resistance. Apoptosis and autophagy were involved in the chemo-resistance to glioblastoma (13). Most recently, pyroptosis, an inflammatory form of programmed cell death, was shown to mediate the anti-tumor immune responses and the resistant mechanism of BRAF and MEK inhibitors for melanoma (14, 15). Furthermore, pyroptosis was involved in programmed death-ligand 1 (PD-L1) translocation and the response to PD-1 blockades (16). These evidences suggested the potential association of autophagy, apoptosis and pyroptosis with therapy responsiveness. However, the prognostic value of autophagy, apoptosis and pyroptosis for HNSCC has not been well developed.

To this end, we aimed to identify the association of autophagy, apoptosis and pyroptosis-related genes with the prognosis of HNSCC, and construct a prognostic signature to predict the outcomes and the responses to radiation, chemotherapy and immunotherapy of HNSCC, especially of HPV negative HNSCC. To be noted, we also assessed the immune landscape of TME and the functional enrichments between the subgroups with distinct gene signatures. Furthermore, we validated the baseline and induced expressions of autophagy, apoptosis and pyroptosis-related genes in two HPV negative HNSCC cell lines.

## Materials and methods

### Patients and datasets

Gene expression information based on RNA sequencing (RNA-seq) data of The Cancer Genome Atlas (TCGA)-HNC dataset was downloaded from UCSC Xena (https://xenabrowser.net/datapages/). The clinicopathological and follow-up information of TCGA-HNC was obtained from Genomic Data Commons (GDC, https://portal.gdc.cancer.gov/). The follow-up information was updated based on Liu et al.’s study (17). HPV status was determined based on Cao et al.’s study (18). HPV-supporting reads over 100 were defined as HPV positivity. Patients with histories of prior malignancies and/or preoperative adjuvant therapies were excluded. Patients without follow-up, TNM stage or HPV status information were also excluded. At last, a total of 379 HNSCC samples was used for the study cohort. Two publicly available datasets (GSE42743 and GSE65858) profiled by microarray were downloaded from Gene Expression Omnibus (GEO) database (https://www.ncbi.nlm.nih.gov/geo/) for further validation. A total of 28 HNSCC samples from Qilu Hospital of Shandong University were also used for validation. The included patients were diagnosed from March 2010 to March 2015, and followed up until May 2019. Clinicopathological features of HNSCC patients in above datasets were shown in **Supplemental Tables 1-4**. The study design was presented in **Figure S1**. This research was approved by the Ethics Committee of Qilu Hospital of Shandong University.

### Human autophagy, apoptosis and pyroptosis-related gene sets

A total of 273 apoptosis-related genes and 153 autophagy-related genes were obtained from Gene Set Enrichment Analysis (GSEA) website (https://www.gsea-msigdb.org/gsea/index.jsp) and a total of 42 pyroptosis-related genes were obtained from GSEA and Gene Ontology (GO) database (http://geneontology.org/). These gene sets were pooled to obtain a combined gene set (n = 434). Ten genes with low expression levels (FPKM = 0) in half or more than half of all HNSCC samples from TCGA dataset were excluded. A total of 424 autophagy, apoptosis and pyroptosis-related genes were used in the following analyses. Above gene sets were shown in **Supplemental Table 5**.

### RNA extraction and sequencing

The RNA extraction and sequencing of above 28 samples from Qilu Hosipital of Shandong University was shown in **Supplementary Methods and Materials**. The sequencing data can be found in Gene Expression Omnibus (GEO) database with the accession number GSE208576 (https://www.ncbi.nlm.nih.gov/geo/query/acc.cgi?acc=GSE208576).

### Construction of the cell death index

On the basis of the RNA-seq data of HNSCC samples obtained from TCGA, overall survival associated genes were screened using univariate Cox regression (*P* < 0.05, log-rank test). The least absolute shrinkage and selection operator (LASSO) regression model was performed to identify the significantly associated genes using R package glmnet (version 4.1.0). The genes significantly affecting overall survival were selected using a forward and backward variable selection procedure using R package MASS (version 4.1.0). At last, the indices of autophagy, apoptosis and pyroptosis and the combined cell death index (CDI) were constructed based on the risk score calculated as follows:


$$\text{Risk score}=\sum_{\text{i}=1}^{\text{n}}\exp_{\text{i}}*\beta_{i}$$

In the above formula, β and exp represent the coefficient and the expression level of the corresponding gene in the multivariate Cox regression model, respectively. The coefficients of the genes to construct respective indices were shown in **Supplemental Table 6**.

### Estimation of immune cell type fractions

The immune cell type fractions were identified by estimating the relative subsets of RNA transcripts utilizing Tumor Immune Estimation Resource (TIMER2.0) (http://timer.cistrome.org/) (19). A deconvolution algorithm of CIBERSORT was applied to quantify the proportions of immune cell types (20). XCELL was applied to evaluate the immune scores and microenvironment scores (21).

### Differential gene expression and functional enrichment analysis

Differentially expressed genes between subgroups were identified using R package DESeq2 (version 4.1.0). Fold-changes (FCs) were calculated. The genes with adjusted *P* < 0.05 and |log_2_FC| > 1.0 were defined as significantly differential genes. Gene Ontology (GO) on biological processes and Kyoto Encyclopedia of Genes and Genomes (KEGG) functional enrichment analyses were performed using R package clusterProfiler (version 4.1.0). GO terms and KEGG pathways with *P* < 0.05 were considered significant.

### Cell culture and treatment

Human HPV negative HNSCC cell lines, CAL27 and SCC-15, were purchased from American Type Culture Collection (ATCC). Cells were cultured in DMEM (Gibco, USA), supplemented with 10% FBS (Gibco, USA). Cells were treated with 10μM chloroquine (CQ) (Sigma, USA) or PBS for 24 h. Certain group of above cells were furtherly treated with 8 Gy radiation and cultured for 24 h described in our previous study (22). Radiation was carried out using Varian 23EX 554 accelerator radiation platform in Department of Radiation Oncology of Qilu Hospital of Shandong University. The required dose, 8 Gy, was calculated according to 6MV X-ray PDD table of Varian 23EX 554 accelerators. The vertical irradiation field was 20 cm × 20 cm.

### Quantitative real time-PCR

Total RNA from cell was extracted using total RNA extraction kit (Fastagen, China) and first strand cDNA was synthesized with a reverse transcription kit (Accurate Biology, China). Quantitative real time-PCR (qRT-PCR) was performed using SYBR Green Master Mix (Accurate Biology, China) in CFX Connect PCR instrument (Bio-Rad laboratories, USA). The primer sequences were listed in **Supplemental Table 7**.

### Apoptosis assay

Apoptosis assay was assessed using the Annexin V-FITC/PI apoptosis detection kit (Vazyme, China) according to the manufacturer’s instructions. The cells were analyzed using a flow cytometer, FACSCalibur (BD, USA).

### Cell proliferation assay

Cells with a density of 4 × 10^4^/ml were seeded in a 96-well plate and incubated for 24 h before being treated with CQ and/or radiation. Cell viability was measured using Cell Counting Kit 8 (CCK-8, Elabscience, China) and determined at 450nm using a microplate reader (INFINITE M200, TECAN, China).

### Western blot

Cells were lysed using RIPA lysis buffer (Beyotime, China). Protein samples were electrophoresed in 12% sodium dodecyl sulfate polyacrylamide gel electrophoresis (SDS-PAGE) and then transferred onto polyvinylidene difluoride membranes (Millipore). After blocking with TBST containing 5% nonfat milk, membranes were incubated with primary antibodies against LC3 (Abcam, UK), p62 (Abcam, UK), caspase-3 (Cell signaling technology, USA), GAPDH (Cell signaling technology, USA), bcl-2 (ABclonal, China) and β-tubulin (ABclonal, China) at 4°C overnight. Then, the membranes were incubated with horseradish peroxidase (HRP) conjugated secondary antibodies (Beyotime, China) for 1 h, and detected by Tanon Image Lab Software.

### Statistical analysis

Patients were divided into high and low risk subgroups according to the cutoff value of 5.938 using R package survminer (version 4.1.0). Time-dependent receiver operating characteristic curve (ROC) analyses were used to evaluate the predictive accuracy. Univariate and multivariate Cox regression analyses were used to identify the prognostic markers. Survival analysis was performed using Kaplan-Meier analysis method and log-rank test. A nomogram was constructed according to the multivariate Cox regression model using R package rms (version 4.1.0). Wilcoxon test, Student’s t test, chi-square test and Fisher’s exact test were used to assess the differences between groups as indicated. Statistical analyses were performed using R software (version 4.1.0) or SPSS (version 26.0) in this study. A two-sided *P* < 0.05 was considered statistically significant.

## Results

### Autophagy, apoptosis and pyroptosis-related genes associated with overall survival of HNSCC

**Figure 1:**
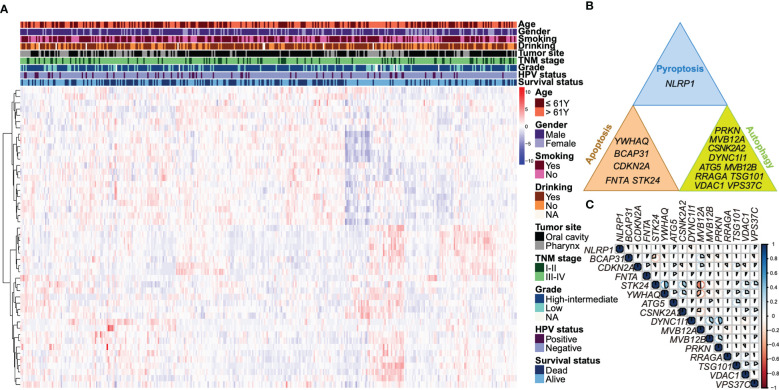
Identification of overall survival associated autophagy, apoptosis and pyroptosis-related genes. **(A)** A heatmap illustrates a unsupervised hierarchical clustering of the expressions of the 48 overall survival associated genes identified by univariate Cox regression based on the Ward D2 method. The data was scaled by row using Z-score normalization. The clinicopathologic characteristics, including age, gender, smoking status, drinking status, tumor site, TNM stage, tumor grade, HPV status, as well as survival status are annotated. **(B)** The 16 overall survival associated autophagy, apoptosis and pyroptosis-related genes are shown respectively. **(C)** A correlation heatmap shows the coefficients of 16 autophagy, apoptosis and pyroptosis-related genes. The area of the pie represents the absolute value of the coefficients. Blue indicates a positive correlation and red is a negative correlation.

We screened 48 overall survival associated autophagy, apoptosis and pyroptosis-related genes using univariate Cox regression analysis in TCGA dataset (**Supplemental Table 8**). Characteristics related to the clinicopathologic characteristics of above genes were displayed in **Figure 1A**. A total of 27 genes were selected using LASSO regression model (**Figure S2**) and 16 genes were selected using stepwise multivariate Cox regression model. The 16 overall survival associated genes comprise five apoptosis-related genes (*BCAP31*, *CDKN2A*, *FNTA*, *STK24* and *YWHAQ*), ten autophagy-related genes (*ATG5*, *CSNK2A2*, *DYNC1I1*, *MVB12A*, *MVB12B*, *RRAGA*, *TSG101*, *PRKN*, *VDAC1* and *VPS37C*) and a pyroptosis-related gene (*NLRP1*) (**Figure 1B**). We subsequently analyzed the correlations of these overall survival associated genes (**Figure 1C**). *STK24* was positively correlated with *YWHAQ* and negatively correlated with *MVB12A*, and *DYNC1I1* was positively correlated with *PRKN* with an absolute coefficient over 0.4.

### Cell death index is an independent prognostic indicator for overall survival of HNSCC

**Figure 2:**
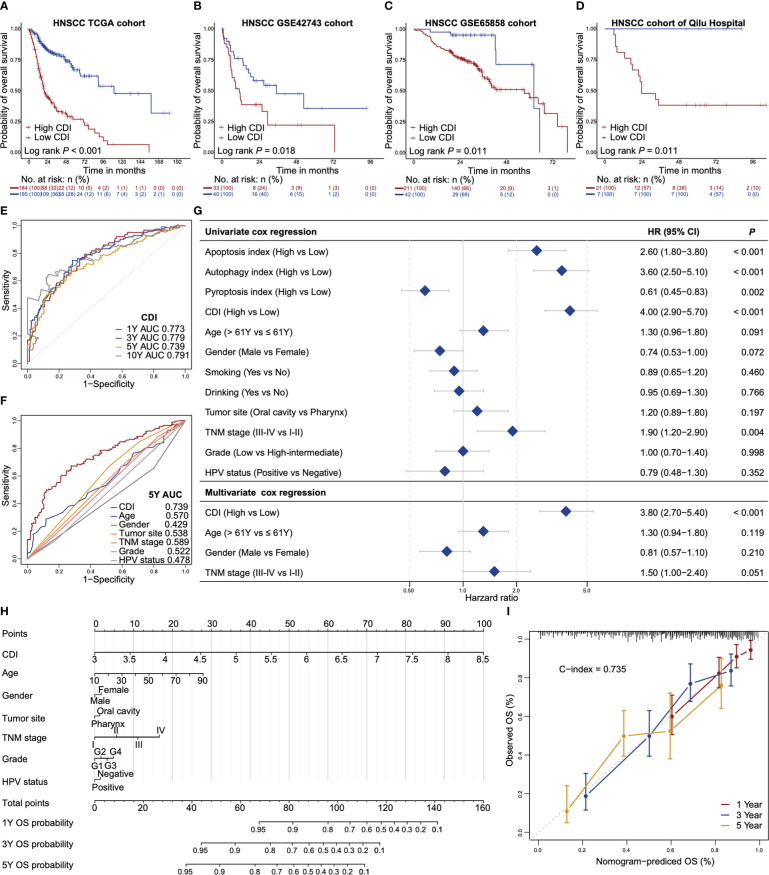
CDI is an independent prognostic indicator for overall survival of HNSCC. **(A–D)** Kaplan-Meier survival curves of high CDI and low CDI subgroups in TCGA dataset **(A)**, GSE42743 dataset **(B)**, GSE65858 dataset **(C)** and the dataset of Qilu Hospital of Shandong University **(D)**. Log-rank test is used to assess the statistical significance. **(E)** ROC curves of CDI for the prediction of overall survival at one-year, three-year, five-year and ten-year. **(F)** ROC curves of CDI and clinicopathological factors for prediction of five-year overall survival. **(G)** Forest plots of the univariate and multivariate Cox regressions of CDI, indices of apoptosis, autophagy and pyroptosis and the clinicopathologic characteristics for overall survival. **(H)** A nomogram estimates the probabilities of one-year, three-year, five-year overall survival for HNSCC patients based on CDI and clinicopathologic characteristics. **(I)** Calibration plots of the nomogram in terms of agreement between the predicted and observed one-year, three-year and five-year overall survival. The close-ended vertical lines indicate the 95% CI, the dashed line represents the ideal performance of the nomogram. HNSCC: head and neck squamous cell carcinoma; CDI: cell death index; ROC: receiver operator characteristic curve; AUC: areas under the curve; TNM: tumor-node-metastasis; HPV: human papillomavirus; HR: hazard ratios; CI: confidential interval; C-index: Harrell’s concordance index using “Hmisc” package.

Based on 16 overall survival associated genes, we established respective indices of autophagy, apoptosis and pyroptosis and the combined cell death index (CDI). HNSCC patients with high CDI scores showed worse overall survivals (median 20 months with 95% confidential interval [CI] of 18 to 29 months) compared to those with low CDI scores (median 110 months with 95% CI of 90 months to not reached) in TCGA dataset (**Figure 2A**). The finding was further validated in GEO datasets (GSE42743 and GSE65858) and the dataset of Qilu Hospital of Shandong University (**Figures 2B** **–D**). Similar trends were found that patients with high CDI scores exhibited worse overall survivals compared to those with low CDI scores (median 11 months vs 35 months in GSE42743, 57 months vs 62 months in GSE65858 and 24 months vs not reached in Qilu Hospital cohort, respectively).

Time-dependent ROC analysis showed that the CDI had an excellent performance to predict one-year, three-year, five-year and ten-year overall survivals with area under ROC curves (AUCs) of 0.773, 0.779, 0.739 and 0.791, respectively (**Figure 2E**). The AUCs of autophagy index, apoptosis index and pyroptosis index and 16 overall survival associated genes were lower than those of CDI (**Figure S3**). Furthermore, the AUC of CDI for five-year overall survival prediction was higher than other potential survival associated factors including age, gender, tumor site, TNM stage, tumor grade, and HPV status (**Figure 2F**). In univariate Cox regression model, CDI, apoptosis index and autophagy index associated with poor prognoses, whereas, pyroptosis index associated with a favorable prognosis (hazard ratio [HR] 4.00, 95% CI 2.90-5.70, *P* < 0.001 for CDI, HR 2.60 for apoptosis index, HR 3.60 for autophagy index and HR 0.61 for pyroptosis index, respectively, **Figure 2G**). In multivariate Cox regression model, CDI was an independent prognostic indicator for overall survival after adjusting for age, gender and TNM stage (HR 3.80, 95% CI: 2.70-5.40, *P* < 0.001, **Figure 2G**).

We also developed a nomogram based on a multivariate Cox model to predict the probabilities of one-year, three-year and five-year overall survival for HNSCC patients using TCGA dataset (**Figure 2H**). The nomogram showed that CDI was the largest contributor, followed by age, TNM stage and tumor grade. The observed and the nomogram-predicted overall survival curves were well-aligned in the calibration plot (**Figure 2I**). Our nomogram model demonstrated a reliable performance with a concordance index (C-index) of 0.735.

### CDI exhibited stronger prognostic effects on HPV negative HNSCC patients, and high CDI HNSCC patients receive survival benefits from radiotherapy

**Figure 3:**
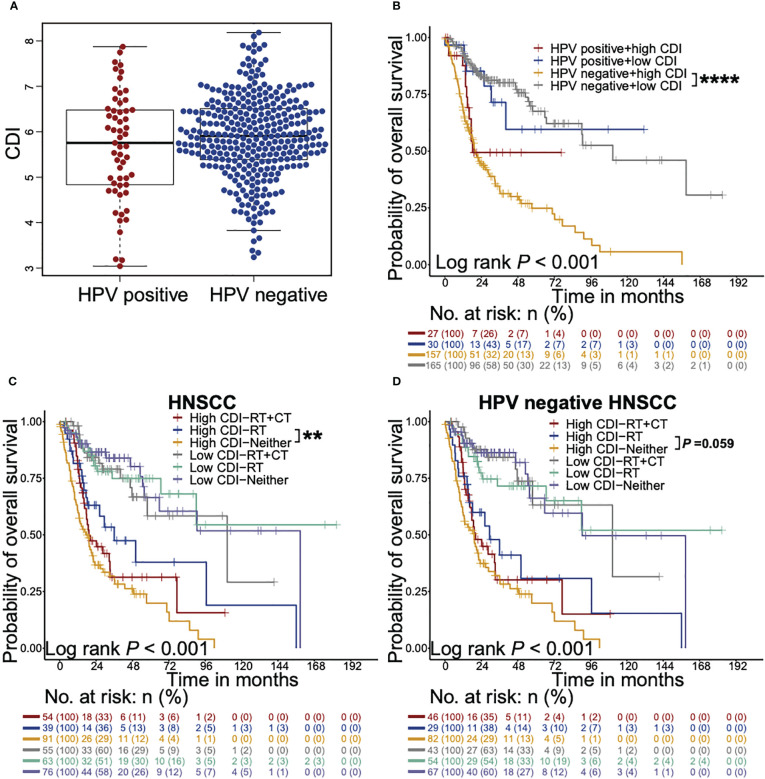
CDI shows stronger prognostic effects on HPV negative HNSCC patients and associates with survival benefits from radiotherapy. **(A)** Beeswarm plot shows the differences of CDI distribution between HPV positive and HPV negative patients in TCGA cohort. The statistical significance is assessed by Wilcoxon test. **(B)** Kaplan-Meier survival curves illustrate the overall survival probabilities of HNSCC patients stratified according to CDI scores and HPV status (**** *P* < 0.0001, log-rank test). **(C, D)** Kaplan-Meier survival curves illustrate the overall survival probabilities of patients with different CDI scores receiving chemoradiotherapy, radiotherapy alone or neither of them in HNSCC patients **(C)** and HPV negative HNSCC subgroup **(D)** (** *P* < 0.01, log-rank test). HNSCC: head and neck squamous cell carcinoma; CDI: cell death index; HPV: human papillomavirus; RT: radiotherapy; CT: chemotherapy. ** *P* < 0.01, **** *P* < 0.0001.

We further investigated the effects of CDI on overall survival across all prespecified subgroups, as defined according to stratified baseline factors and other factors known to affect survivals using TCGA dataset (**Figures 3A, B**, **S4**). Similar trends were found in all subgroups, except for HPV positive HNSCC patients. This interesting finding addressed our focus on the stratification of HPV status, especially the majority of HNSCC, HPV negative patients.

To evaluate whether HNSCC patients with high or low CDI scores obtained different survival benefits from radiotherapy or chemoradiotherapy, we analyzed the overall survival probabilities of the high and low CDI HNSCC patients stratified according to adjuvant therapies using TCGA dataset (**Figures 3C, D** **)**. Only one patient who received chemotherapy alone was exclude in the following analysis. HNSCC patients receiving radiotherapy showed favorable overall survivals in high CDI subgroup, but not in low CDI subgroup (*P* = 0.009 and *P* = 0.938, respectively, **Figure 3C**). Notably, similar trends were found in HPV negative subgroup (*P* = 0.059, **Figure 3D**) and HPV positive patients (*P* = 0.045, **Figure S5A**). Taken together, these findings suggested that although HPV negative patients with high CDI scores exhibited the poorest outcomes, they might still receive overall survival benefits from radiotherapy.

### Low CDI HNSCC patients exhibit active immune microenvironment in HPV negative HNSCC subgroup

**Figure 4:**
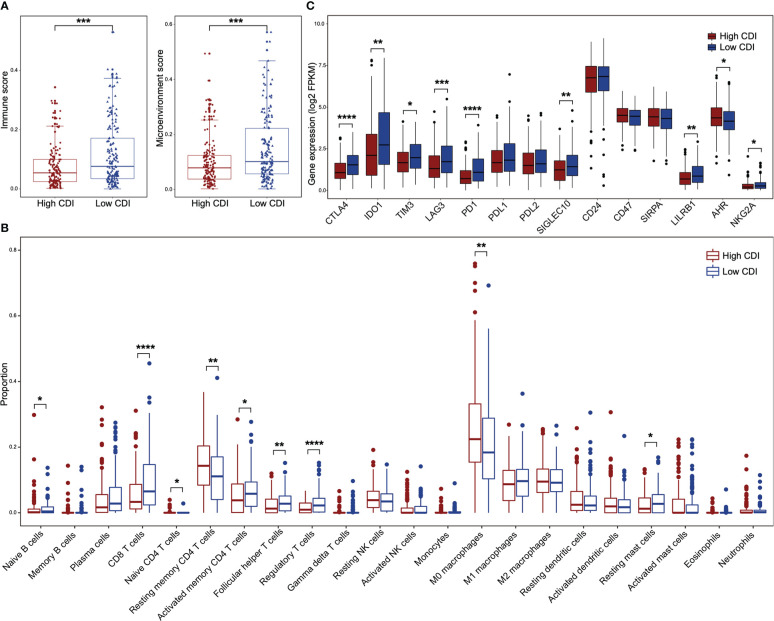
The immune landscape between high and low CDI HNSCC in HPV negative subgroup. **(A)** Boxplots illustrate the immune scores and the microenvironment scores of high and low CDI subgroups in HPV negative HNSCC assessed using XCELL. **(B)** Boxplots of relative proportions of infiltrating immune cells in high and low CDI subgroups in HPV negative HNSCC assessed using CIBERSORT. **(C)** Boxplots of the expressions of immune checkpoint genes in different CDI subgroups in HPV negative HNSCC. HNSCC: head and neck squamous cell carcinoma; CDI: cell death index; HPV: human papillomavirus. Statistical significance is assessed by Wilcoxon test. *P < 0.05, **P < 0.01, ***P < 0.001, ****P < 0.0001.

We evaluated the immune landscape of high and low CDI HNSCC patients using TCGA dataset. HPV negative HNSCC patients with low CDI scores showed higher immune scores and microenvironment scores (**Figure 4A**). However, the differences of above scores were not found between high CDI and low CDI subgroups in HPV positive patients (**Figure S5B**). To evaluate the immune cell infiltration of the HNSCC patients with different CDI scores, we applied algorithms of the deconvolution (**Figure 4B**, **S5C**). The infiltrations of resting memory CD4^+^ T cells and M0 macrophages were higher in high CDI subgroup in both HPV negative and positive HNSCC patients. Whereas, naive B cells, CD8^+^ T cells, activated memory CD4^+^ T cells, follicular helper T cells, regulatory T cells and resting mast cells were more abundant in low CDI subgroup only in HPV negative HNSCC subgroup.

Moreover, to evaluate the potential efficacy of the CDI score for immune checkpoint blockades, we examined the expressions of 14 key immune checkpoint genes between high and low CDI subgroups in HNSCC patients using TCGA dataset. We found that the expression of *CTLA4*, *IDO1*, *TIM3*, *LAG3*, *PD1*, *SIGLEC10*, *LILRB1* and *NKG2A* were increased in low CDI subgroup of HPV negative HNSCC patients (**Figure 4C**), but not in HPV positive HNSCC patients (**Figure S5D**). These suggested that HPV negative patients with low CDI scores might receive potential benefits from checkpoint blockades.

### Low CDI HNSCC patients showed a gene enrichment in immune functions-related pathways in HPV negative HNSCC subgroup

**Figure 5:**
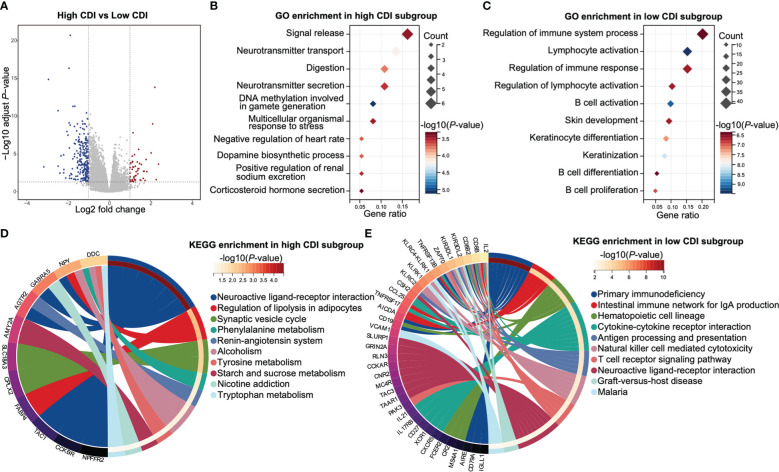
Functional enrichments of HPV negative HNSCC patients with different CDI scores. **(A)** A volcano plot of differentially expressed genes between the subgroups of different CDI scores of HPV negative HNSCC. **(B, C)** The top 10 enriched GO biological processes of up-regulated genes in high CDI subgroup **(B)** and low CDI subgroup **(C)**. **(D, E)** The top 10 enriched KEGG pathways of up-regulated genes in high CDI subgroup **(D)** and low CDI subgroup **(E)**. HNSCC: head and neck squamous cell carcinoma; CDI: cell death index; HPV: human papillomavirus; GO: Gene Ontology; KEGG: Kyoto Encyclopedia of Genes and Genomes.

Considering the differential prognostic effect of CDI for HPV negative and HPV positive patients, as well as the distinct immune microenvironments between high CDI and low CDI subgroups in HPV negative HNSCC patients, we further analyzed the differentially expressed genes of HPV negative HNSCC patients with different CDI scores. A total of 288 differential genes were identified, of which 51 genes were upregulated and 237 genes were downregulated in high CDI subgroup (**Figure 5A** and **Supplemental Table 9**).

GO enrichment analyses revealed that up regulated genes in low CDI subgroup were mainly enriched in immune functions-related pathways, including regulation of immune system process, lymphocyte activation, regulation of immune response, regulation of lymphocyte activation, B cell activation, B cell differentiation and B cell proliferation. However, up regulated genes in high CDI subgroup were rarely enriched in immune functions-related pathways (**Figures 5B, C** and **Supplemental Table 10**).

KEGG enrichment analyses demonstrated that up regulated genes in high CDI subgroup were mainly enriched in metabolism-related pathways, including tryptophan metabolism, starch and sucrose metabolism, tyrosine metabolism, and phenylalanine metabolism. Whereas up regulated genes in low CDI subgroup were mainly enriched in immune functions-related pathways, including cytokine-cytokine receptor interaction, antigen processing and presentation, natural killer cell mediated cytotoxicity and T cell receptor signaling pathways (**Figures 5D, E** and **Supplemental Table 10**).

### The expression of the sixteen overall survival associated genes in two HPV negative HNSCC cell lines

**Figure 6:**
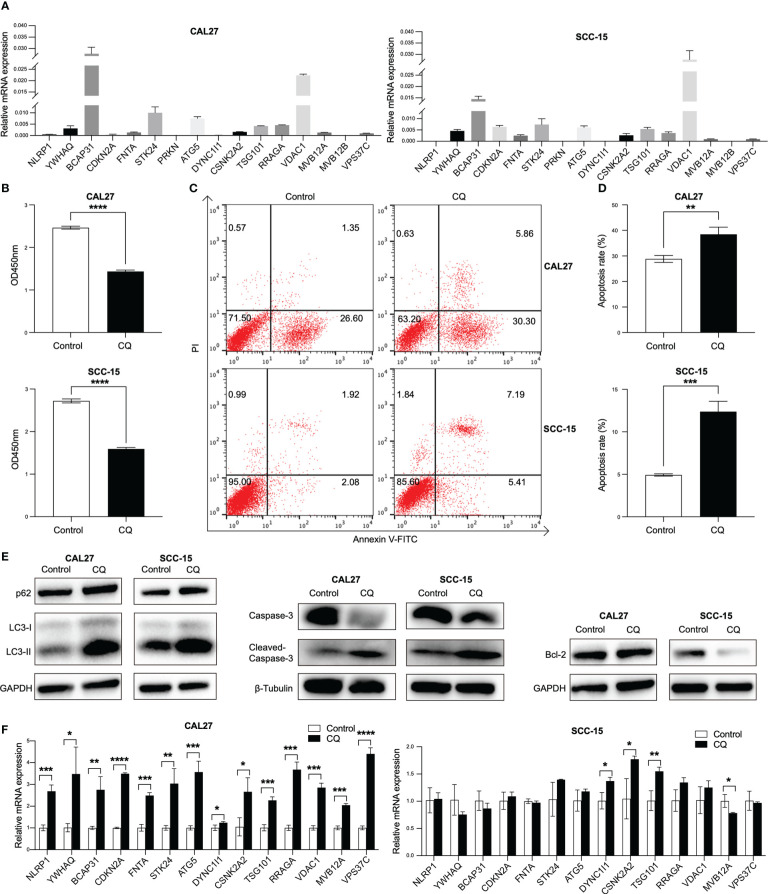
Validation of the expression of 16 overall survival associated genes in two HPV negative HNSCC cell lines. **(A)** The transcripts of the 16 overall survival associated genes in CAL27 and SCC-15 cell lines detected by qRT-PCR. **(B)** Cell proliferation after chloroquine treatment for 24 h in CAL27 and SCC-15 cell lines assessed by CCK8 assay. **(C, D)** Cell apoptosis post chloroquine treatment for 24 h in CAL27 and SCC-15 cell lines indicated by annexin V and PI using flow cytometry. **(E)** The inductions of autophagy-related proteins (LC3 and p62) and apoptosis-related proteins (bcl-2 and caspase-3). **(F)** The transcripts of 14 overall survival associated genes post chloroquine treatment in CAL27 and SCC-15 cell lines. HNSCC: head and neck squamous cell carcinoma; HPV: human papillomavirus; CQ: chloroquine. Each bar represents the mean ± SD. Statistical significance is assessed by Student’s t test. *P < 0.05, **P < 0.01, ***P < 0.001, ****P < 0.0001.

To validate the findings of database analysis, we measured the baseline mRNA expression levels of above 16 overall survival associated genes of two HPV negative HNSCC cells, CAL27 and SCC-15, using qRT-PCR (**Figure 6A**). In response to an autophagy inhibitor, chloroquine (CQ), the cell viabilities were significantly inhibited in both CAL27 and SCC-15 cells (**Figure 6B**). Furthermore, we found that CQ induced apoptosis in both cell lines (**Figures 6C, D** **)**. Autophagy-related proteins (LC3 and p62) was induced and bcl-2 was reduced and caspase-3 was cleaved in response to CQ indicating that CQ might inhibit autophagy and induce apoptosis (**Figure 6E**). Above overall survival associated genes were all induced post CQ treatment in CAL27 cell line, except for two low expression genes (*PRKN* and *MVB12B*). *DYNC1I1*, *CSNK2A2* and *TSG101* were induced and *MVB12A* were reduced post CQ treatment in SCC-15 cell line (**Figure 6F**).

### Radiation induces apoptosis and the expression of *VDAC1* in HPV negative HNSCC cell lines

**Figure 7:**
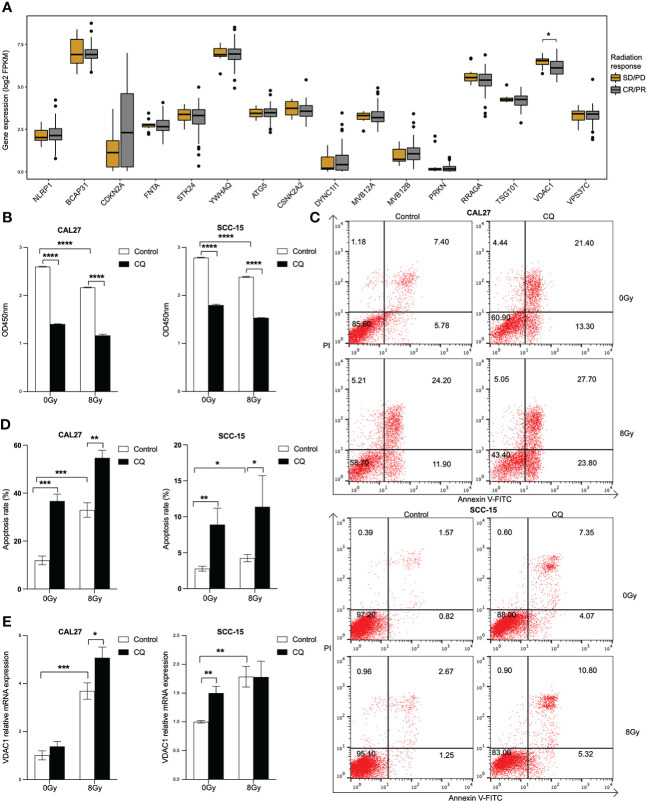
Radiation enhances the expression of *VDAC1* in HPV negative HNSCC cell lines. **(A)** Boxplots of the expressions of 16 overall survival associated genes between radiation sensitive and resistant subgroups in HPV negative HNSCC. **(B)** Cell proliferation after chloroquine treatment for 24 h, following radiation in CAL27 and SCC-15 cell lines. **(C, D)** Cell apoptosis post chloroquine and/or radiation treatments in CAL27 and SCC-15 cell lines indicated by annexin V and PI using flow cytometry. **(E)** *VDAC1* transcript post chloroquine and/or radiation treatments in CAL27 and SCC-15 cell lines. HNSCC: head and neck squamous cell carcinoma; HPV: human papillomavirus; CQ: chloroquine. Each bar represents the mean ± SD. Statistical significance is assessed by Student’s t test. *P < 0.05, **P < 0.01, ***P < 0.001, ****P < 0.0001.

We further identified a differentially expressed gene, *VDAC1*, between radiation sensitive and radiation resistant subgroups in HPV negative HNSCC patients (**Figure 7A**). The expression of *VDAC1* was significantly lower in radiation sensitive subgroup. *In vitro*, cell viabilities were significantly inhibited in both CAL27 and SCC-15 cells lines in response to CQ and/or radiation (**Figure 7B**). We further found that CQ and/or radiation induced apoptosis in both CAL27 and SCC-15 cell lines (**Figures 7C, D** **)**. Furthermore, *VDAC1* was induced post CQ and/or radiation treatments in both CAL27 and SCC-15 cell lines (**Figure 7E**).

## Discussion

The need to predict the prognosis and therapy responsiveness of HNSCC patients is unmet, especially for HPV negative subgroup, the majority and the poor outcome population of HNSCC patients (23). Tumor cell death has been reported to associate with the resistance of radiation, chemotherapy and immunotherapy (24–26). To this end, we identified 16 autophagy, apoptosis and pyroptosis-related genes and constructed a cell death index (CDI), to predict the prognosis of HNSCC. Furthermore, high CDI HNSCC patients received survival benefits from radiotherapy. Low CDI HNSCC patients showed higher checkpoint gene expressions, an inflamed TME and predominant enrichments of immune response-related functions, which were found only in HPV negative subgroup. We further validated the baseline and induced expressions of the 16 genes in two HPV negative HNSCC cell lines.

We established an apoptosis related gene index (*BCAP31*, *CDKN2A*, *FNTA*, *STK24* and *YWHAQ*), and an autophagy related gene index (*ATG5*, *CSNK2A2*, *DYNC1I1*, *MVB12A*, *MVB12B*, *RRAGA*, *TSG101*, *PRKN*, *VDAC1* and *VPS37C*), a pyroptosis related gene index (*NLRP1*), as well as the combined index, cell death index (CDI). Remarkably, the combined index, CDI, demonstrated a significantly improved prognostic value compared with the single type indices. There are several studies reported prognostic models based on cell death related genes (27–33). However, these models were based on certain cell death type and obtained limited predictive potentials. Consistent with our study, *ATG5* and *PRKN* were identified as risk genes (27, 29, 30), whereas, *CDKN2A* and *NLRP1* predicted favorable survivals of HNSCC (28–33). Together, the combined index, CDI, supplied a more promising efficacy for the prognosis of HNSCC.

HPV negative HNSCC patients have poor prognosis and less responsiveness to radiation (9, 11, 34). Consistently, HPV negative HNSCC patients with high CDI scores showed worse overall survivals. However, these patients achieved improved overall survival receiving radiation. On the other hand, HPV negative HNSCC patients were also believed to poorly response to checkpoint blockades (10, 35, 36). We found a subgroup of HPV negative HNSCC patients, low CDI subgroup, showed higher checkpoint gene expressions. Moreover, this subgroup also exhibited an inflamed TME and predominant enrichments of immune response-related functions. An inflammatory TME is generally believed to have the potential to respond to checkpoint blockade (37, 38). Therefore, our findings suggested that HPV negative HNSCC patients with high CDI scores might receive benefits from radiotherapy, whereas, those with low CDI scores might have the potential to achieve favorable outcomes from checkpoint blockades.

*In vitro*, the baseline and the induced expressions of the 16 prognosis associated genes were validated in two HPV negative HNSCC cell lines, CAL27 and SCC-15. The autophagy inhibitor, CQ, inhibited the cell viability and induce the apoptosis in both cell lines. Furthermore, *VDAC1* was highly induced post CQ and/or radiation treatments in both cell lines. Voltage-dependent anion channel 1 (*VDAC1*), the most abundant isomers of VDAC protein family, is a key regulator in aerobic glycolysis and proliferation of cancer cell (39, 40). Studies demonstrated that *VDAC1* was induced post radiation treatment (41, 42). In our study, differently from other overall survival associated autophagy, apoptosis and pyroptosis-related genes, the expression of *VDAC1* was significantly related to radiation response in HPV negative HNSCC. Consistently, *in vitro* experiment showed that radiation induced apoptosis and inhibited cell viabilities of two HPV negative cell lines. Notably, the expression of *VDAC1* was induced in response to radiation in both CAL27 and SCC-15 cell lines.

There are certain limitations of our study. The primary limitation is that it was based on publicly available datasets and a dataset from our retrospective cohort. Thus, further prospective studies for validation might be needed. Next, we only validated the baseline and induced expressions of the 16 survival associated genes in HNSCC cell lines. The mechanisms how these genes affect the tumor biology and progress might need further investigation.

In conclusion, we identified 16 autophagy, apoptosis and pyroptosis-related genes and constructed a cell death index (CDI), the CDI predicted the prognosis of HNSCC patients independently. HNSCC patients with high CDI scores might benefit from radiation, whereas, those with HPV negativity and low CDI scores might receive potential benefit from checkpoint immunotherapy. Our results not only established a prognostic signature, but also emphasized its implements of therapeutic options in HPV negative HNSCC patients, the majority and the poor outcome population of HNSCC.

## Data availability statement

The datasets presented in this study can be found in online repositories. The names of the repository/repositories and accession number(s) can be found in the article/**Supplementary Material**.

## Ethics statement

This study was approved by the Ethics Committee of Qilu Hospital of Shandong University.

## Author contributions

ZN for acquisition, analysis and interpretation of data, statistical analysis and drafting of the manuscript. XQ and YD for study concept and design, technical and material support, obtained funding and study supervision, manuscript revision and editing. KW, SL, ZD, and AC for obtained resources. JS, DA, CM, and YC for data curation and validation. ZM and MN for manuscript editing. All authors contributed to the article and approved the submitted version.

## Supplementary material

Supplementary Figure 1 Flowchart of study design. The flow work of the identification and validation of a prognostic signature of autophagy, apoptosis and pyroptosis-related genes. HNSCC, head and neck squamous cell carcinoma; TNM, tumor-node-metastasis; LASSO, the least absolute shrinkage and selection operator; OS, overall survival; RT, radiotherapy; CT, chemotherapy.

Supplementary Figure 2 Identification of prognostic autophagy, apoptosis and pyroptosis-related genes in HNSCC. The genes are identified through LASSO regression algorithm. **(A)** Partial likelihood deviance reveals by the LASSO regression model in the 10-fold cross validation. The vertical dotted lines were drawn at the optimal values by the minimum criteria. **(B)** LASSO coefficient profiles of 27 selected autophagy, apoptosis and pyroptosis-related genes in the 10-fold cross validation. LASSO, the least absolute shrinkage and selection operator.

Supplementary Figure 3 Time dependent ROC analyses of autophagy, apoptosis and pyroptosis-related genes and indices. Time dependent ROC curves of (*BCAP31, CDKN2A, FNTA, STK24, YWHAQ, ATG5, CSNK2A2, DYNC1I1, MVB12A, MVB12B, RRAGA, TSG101, PRKN, VDAC1, VPS37C* and *NLRP1*) are shown in the top panel. Time dependent ROC curves of the indices of autophagy, apoptosis and pyroptosis are shown in the bottom panel. OS, overall survival; ROC, receiver operator characteristic; AUC, areas under the curve.

Supplementary Figure 4 The predominantly prognostic value of CDI in subgroups of clinical factors. **(A, C, E, G, I, K** and **M)** Beeswarm plots show the differences of CDI distribution between elder and younger patients **(A)**, male and female patients **(C)**, smoking and non-smoking patients **(E)**, drinking and non-drinking patients **(G)**, patients with tumors in oral cavity and those in pharynx **(I)**, patients with TNM stage III-IV and those with TNM stage I-II **(K)**, patients with low grade and those with high-intermediate grade tumors **(M)** in TCGA cohort. The statistical significance is assessed by Wilcoxon test. **(B, D, F, H, J, L** and **N)** Kaplan-Meier survival curves illustrate the overall survival probabilities of HNSCC patients stratified according to CDI scores and above subgroup factors, age **(B)**, gender **(D)**, smoking status **(F)** and drinking status **(H)**, tumor site **(J)**, TNM stage **(L)** and tumor grade **(N)** (** *P* < 0.01, **** *P* < 0.0001, log-rank test). CDI, cell death index; HNSCC, head and neck squamous cell carcinoma; OS, overall survival; TNM, tumor-node-metastasis.

Supplementary Figure 5 Association of CDI with survival benefits of radiation and immune landscape in HPV positive HNSCC patients. **(A)** Kaplan-Meier survival curves illustrate the overall survival probabilities of HNSCC patients with different CDI scores receiving chemoradiotherapy, radiotherapy alone or neither of them in HPV positive HNSCC patients (* *P* < 0.05, log-rank test). **(B)** Boxplots illustrate the immune scores and the microenvironment scores of high and low CDI subgroups in HPV positive HNSCC assessed using XCELL. **(C)** Boxplots of relative proportions of infiltrating immune cells in high and low CDI subgroups in HPV positive HNSCC assessed using CIBERSORT. **(D)** Boxplots of the expressions of immune checkpoint genes in different CDI subgroups in HPV positive HNSCC. The statistical significance is assessed by Wilcoxon test. * *P* < 0.05, ** *P* < 0.01. HNSCC, head and neck squamous cell carcinoma; CDI, cell death index; HPV, human papillomavirus; RT, radiotherapy; CT, chemotherapy.
