# Supplementary figures and images for: Identification and validation of a prognostic signature of autophagy, apoptosis and pyroptosis-related genes for head and neck squamous cell carcinoma: to imply therapeutic choices of HPV negative patients
Source: Front Immunol. 2023 Jan 10;13:1100417. doi: 10.3389/fimmu.2022.1100417 (PMC9872116; doi:10.3389/fimmu.2022.1100417)

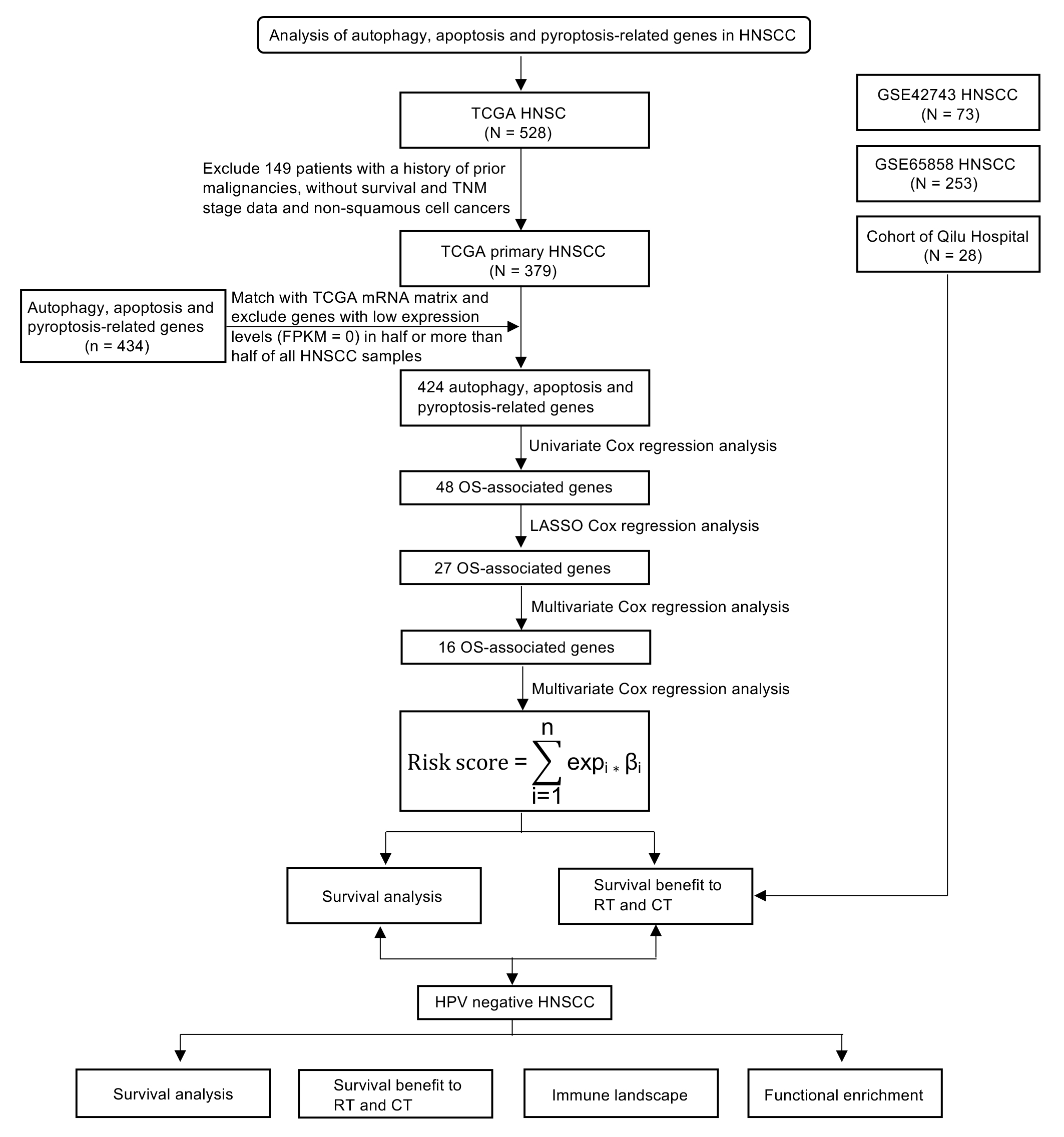

Supplement: Supplementary Figure 1 — Flowchart of study design. The flow work of the identification and validation of a prognostic signature of autophagy, apoptosis and pyroptosis-related genes. HNSCC, head and neck squamous cell carcinoma; TNM, tumor-node-metastasis; LASSO, the least absolute shrinkage and selection operator; OS, overall survival; RT, radiotherapy; CT, chemotherapy. [file Image_1.tiff]

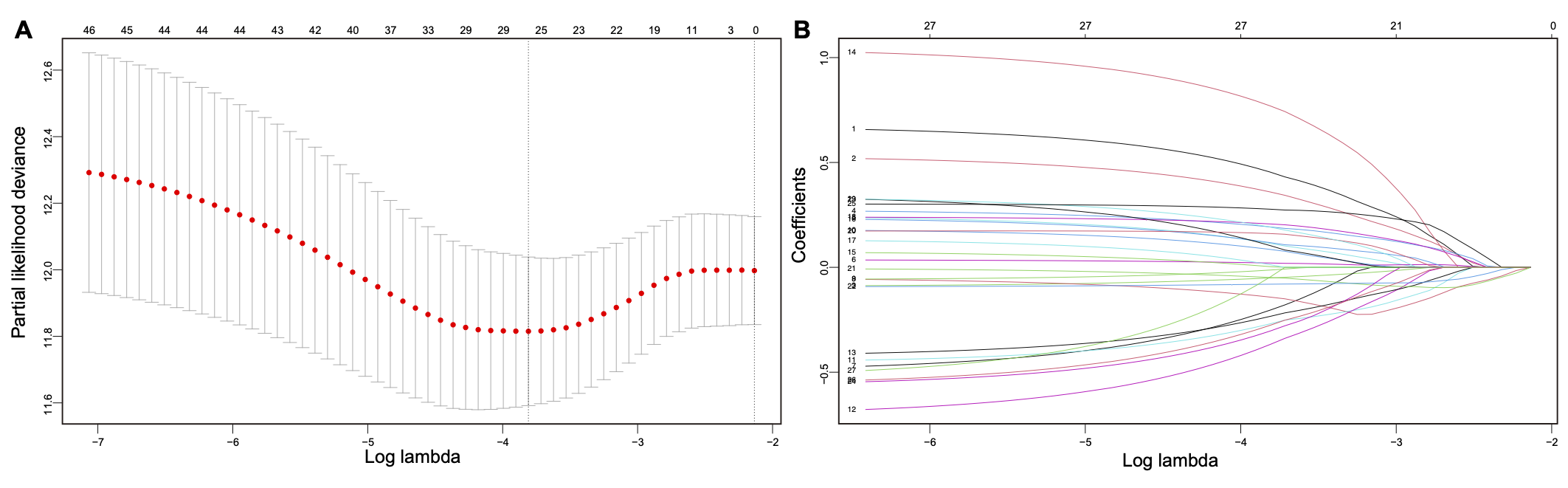

Supplement: Supplementary Figure 2 — Identification of prognostic autophagy, apoptosis and pyroptosis-related genes in HNSCC. The genes are identified through LASSO regression algorithm. (A) Partial likelihood deviance reveals by the LASSO regression model in the 10-fold cross validation. The vertical dotted lines were drawn at the optimal values by the minimum criteria. (B) LASSO coefficient profiles of 27 selected autophagy, apoptosis and pyroptosis-related genes in the 10-fold cross validation. LASSO, the least absolute shrinkage and selection operator. [file Image_2.tiff]

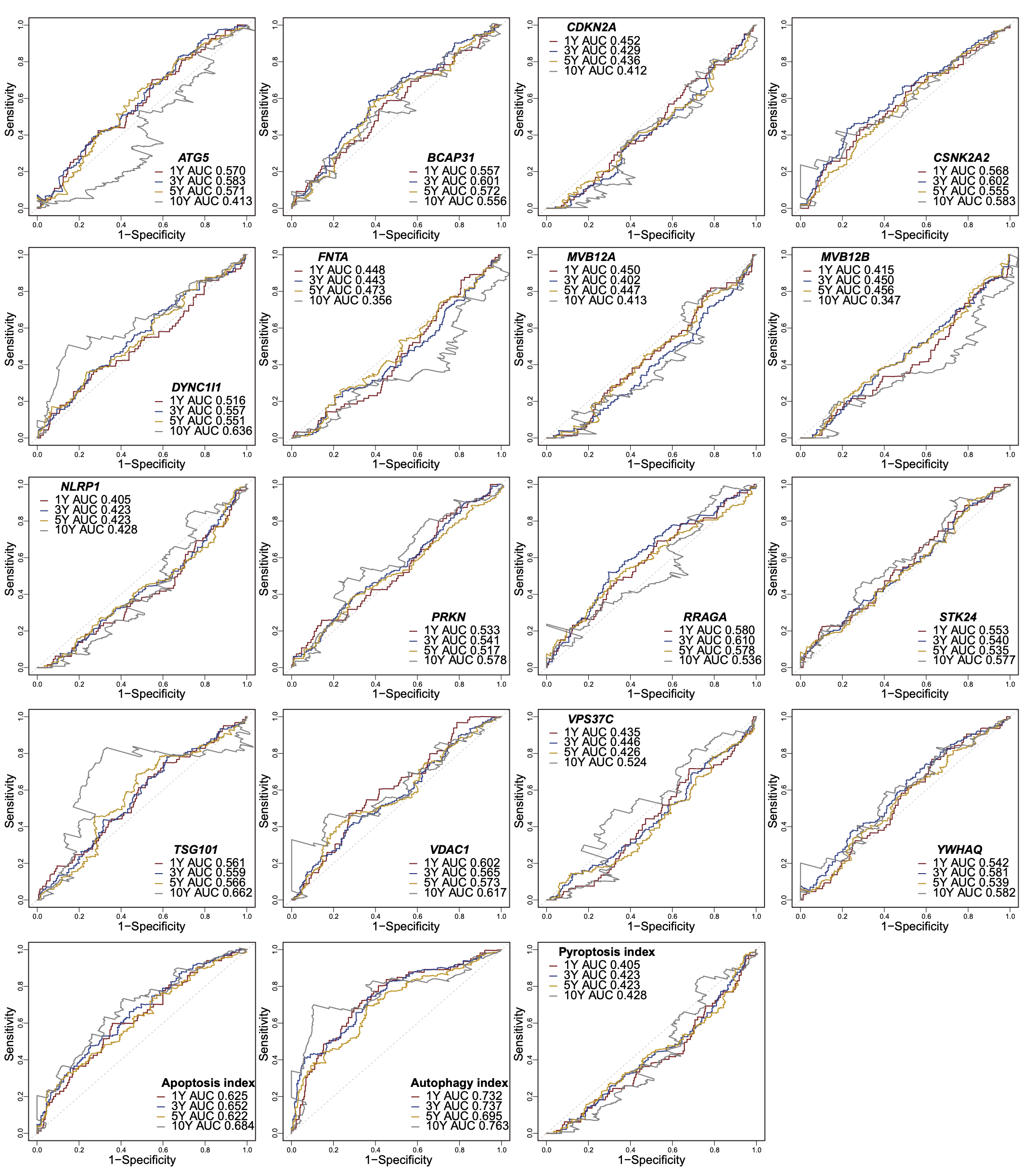

Supplement: Supplementary Figure 3 — Time dependent ROC analyses of autophagy, apoptosis and pyroptosis-related genes and indices. Time dependent ROC curves of (BCAP31, CDKN2A, FNTA, STK24, YWHAQ, ATG5, CSNK2A2, DYNC1I1, MVB12A, MVB12B, RRAGA, TSG101, PRKN, VDAC1, VPS37C and NLRP1) are shown in the top panel. Time dependent ROC curves of the indices of autophagy, apoptosis and pyroptosis are shown in the bottom panel. OS, overall survival; ROC, receiver operator characteristic; AUC, areas under the curve. [file Image_3.tiff]

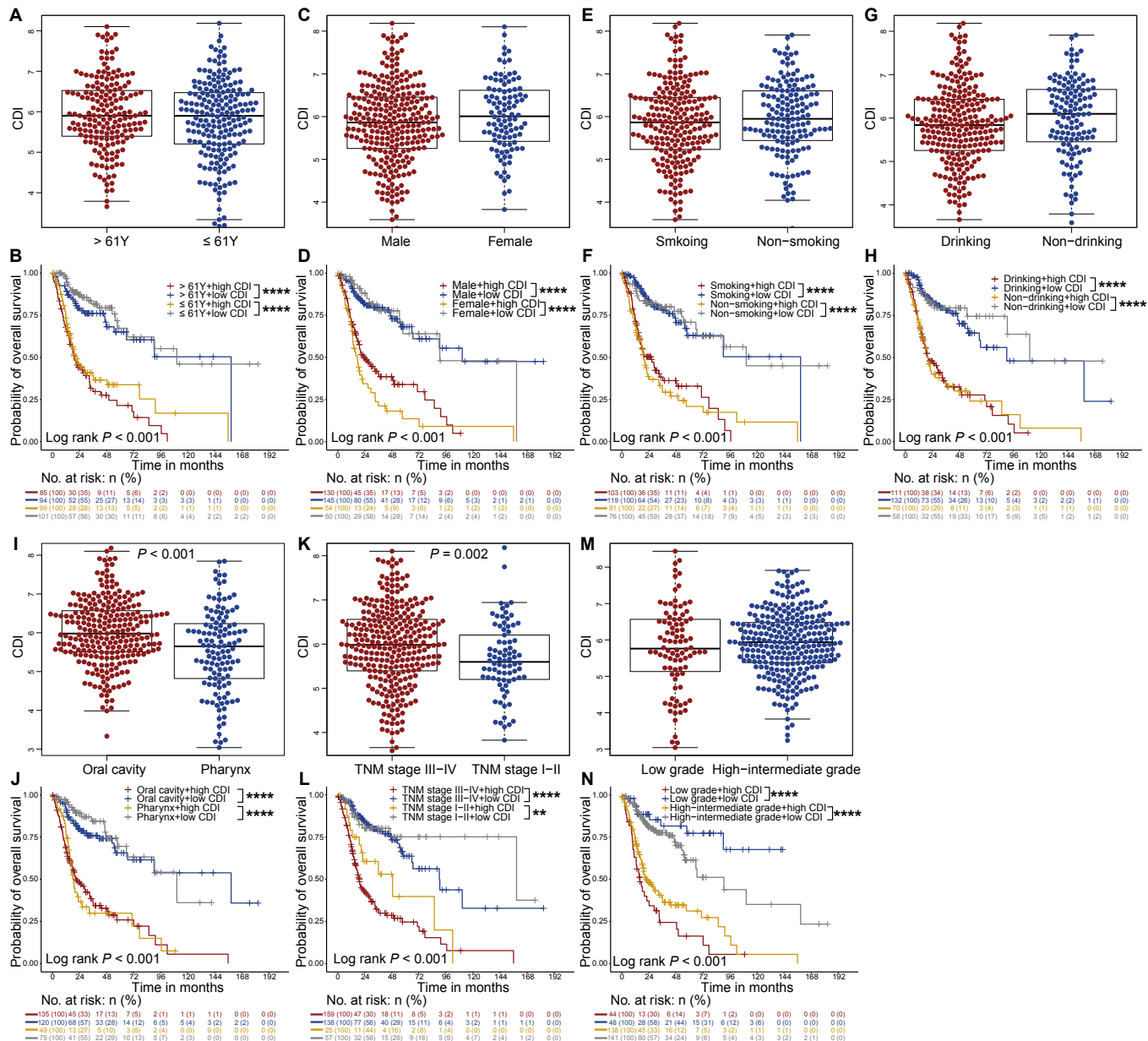

Supplement: Supplementary Figure 4 — The predominantly prognostic value of CDI in subgroups of clinical factors. (A, C, E, G, I, K and M) Beeswarm plots show the differences of CDI distribution between elder and younger patients (A), male and female patients (C), smoking and non-smoking patients (E), drinking and non-drinking patients (G), patients with tumors in oral cavity and those in pharynx (I), patients with TNM stage III-IV and those with TNM stage I-II (K), patients with low grade and those with high-intermediate grade tumors (M) in TCGA cohort. The statistical significance is assessed by Wilcoxon test. (B, D, F, H, J, L and N) Kaplan-Meier survival curves illustrate the overall survival probabilities of HNSCC patients stratified according to CDI scores and above subgroup factors, age (B), gender (D), smoking status (F) and drinking status (H), tumor site (J), TNM stage (L) and tumor grade (N) (** P < 0.01, **** P < 0.0001, log-rank test). CDI, cell death index; HNSCC, head and neck squamous cell carcinoma; OS, overall survival; TNM, tumor-node-metastasis. [file Image_4.pdf]

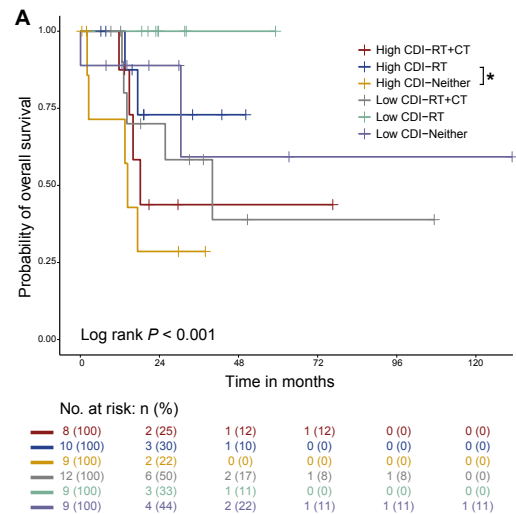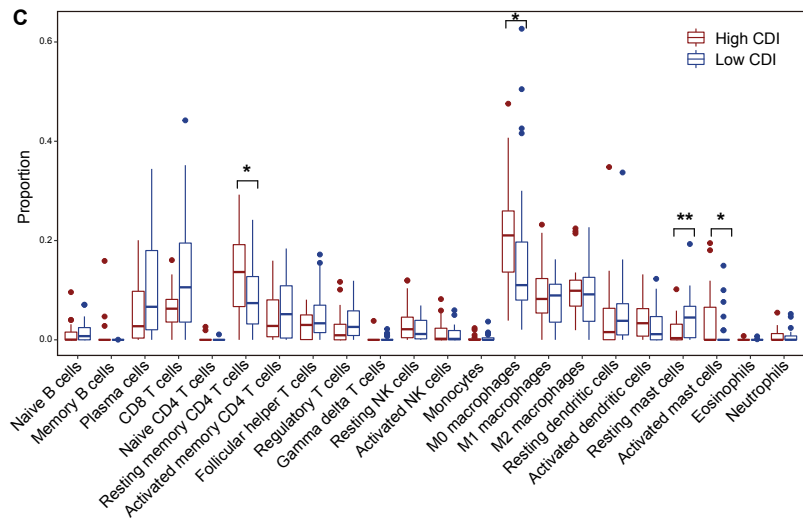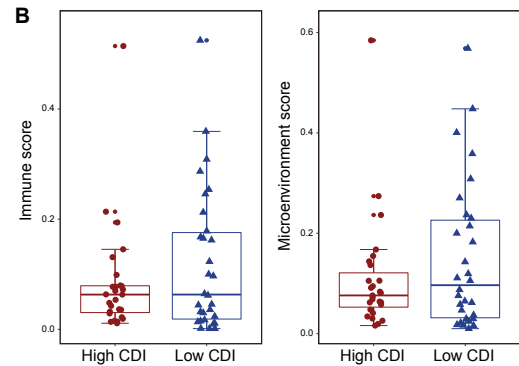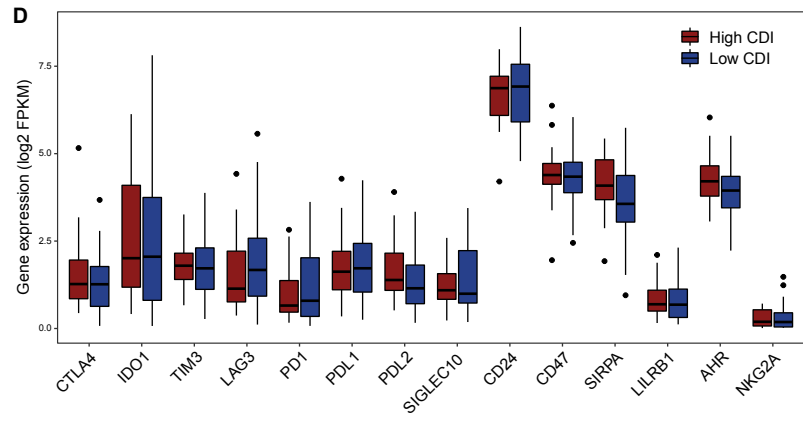

Supplement: Supplementary Figure 5 — Association of CDI with survival benefits of radiation and immune landscape in HPV positive HNSCC patients. (A) Kaplan-Meier survival curves illustrate the overall survival probabilities of HNSCC patients with different CDI scores receiving chemoradiotherapy, radiotherapy alone or neither of them in HPV positive HNSCC patients (* P < 0.05, log-rank test). (B) Boxplots illustrate the immune scores and the microenvironment scores of high and low CDI subgroups in HPV positive HNSCC assessed using XCELL. (C) Boxplots of relative proportions of infiltrating immune cells in high and low CDI subgroups in HPV positive HNSCC assessed using CIBERSORT. (D) Boxplots of the expressions of immune checkpoint genes in different CDI subgroups in HPV positive HNSCC. The statistical significance is assessed by Wilcoxon test. * P < 0.05, ** P < 0.01. HNSCC, head and neck squamous cell carcinoma; CDI, cell death index; HPV, human papillomavirus; RT, radiotherapy; CT, chemotherapy. [file Image_5.pdf]
