# Supplementary material for: Identification and validation of a prognostic signature of autophagy, apoptosis and pyroptosis-related genes for head and neck squamous cell carcinoma: to imply therapeutic choices of HPV negative patients
Source: Front Immunol. 2023 Jan 10;13:1100417. doi: 10.3389/fimmu.2022.1100417 (PMC9872116; doi:10.3389/fimmu.2022.1100417)
Supplement: Supplementary file 6 [file DataSheet_1.docx]

Supplementary Methods and Materials

# RNA extraction and sequencing

Total RNA from paraffin-embedded tumor tissue was isolated using RecoverAll^TM^ Total Nucleic Acid Isolation Kit (Invitrogen, USA) according to the manufacturer’s instructions. The concentration and the quantity of RNA samples were determined using Qubit^®^3.0 Fluorometer (Life Technologies, USA) and NanoDrop One spectrophotometer (Thermo Fisher Scientific Inc., USA). One microgram of RNA was used for the RNA sample preparations. RNA-seq strand-specific libraries were constructed using VAHTS^®^ Total RNA-seq (H/M/R) Library Prep Kit (Vazyme, China) according to the manufacturer’s instructions. Briefly, RNA was purified by magnetic beads after rRNA removal. Then the purified RNA was fragmented into small pieces using divalent cations for 8 min at 94°C. The cleaved RNA fragments were copied into the first strand cDNA using reverse transcriptase and random primers. Second strand cDNA synthesis was subsequently performed using DNA polymerase I and RNase H. These cDNA fragments were processed for the end repair process, addition of a single ‘A’ base, and ligation of the adapters. The products were purified and enriched by PCR to create the final cDNA library. Purified libraries were quantified and validated by Qubit^®^ 3.0 Fluorometer and Agilent 2100 bioanalyzer to confirm the insert size and the concentration. The cluster was generated by cBot with the library diluted to 10 pM and then were sequenced on Illumina NovaSeq 6000 platform (Illumina, USA). The library construction and sequencing were performed by Sinotech Genomics Co., Ltd (Shanghai, China).

Raw sequencing files were converted to FASTQ files using base calling analysis. FastQC (version 0.11.7) was used for quality control metrics (<https://www.bioinformatics.babraham.ac.uk/projects/fastqc/>). The FASTQ files were filtered using fastp (<https://github.com/OpenGene/fastp>)^1^. Reads were mapped to the reference genome (GRCh38) using HISAT2 (Hierarchical Indexing for Spliced Alignment of Transcripts)^2,3^. SAM (sequencing alignment/map) files were sorted and converted to BAM (binary alignment/map) files using SAMtools^4^. StringTie was then used to quantify the abundance of the transcripts and genes^5^. TMM (trimmed mean of M values) algorithm (<https://www.ncbi.nlm.nih.gov/research/cog-project/>) was used for normalization. Fragments per kilobase of transcript per million mapped reads (FPKM) was applied to evaluate the expression levels of individual genes, which was calculated as follows:


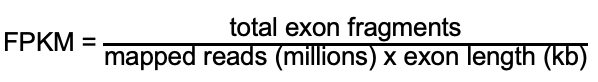


**REFERENCES**

1. Chen S, Zhou Y, Chen Y, Gu J. fastp: an ultra-fast all-in-one FASTQ preprocessor. *Bioinformatics.* 2018;34(17):i884-i890.

2. Buchfink B, Xie C, Huson DH. Fast and sensitive protein alignment using DIAMOND. *Nat Methods.* 2015;12(1):59-60.

3. Kim D, Langmead B, Salzberg SL. HISAT: a fast spliced aligner with low memory requirements. *Nat Methods.* 2015;12(4):357-360.

4. Li H, Handsaker B, Wysoker A, et al. The Sequence Alignment/Map format and SAMtools. *Bioinformatics.* 2009;25(16):2078-2079.

5. Pertea M, Pertea GM, Antonescu CM, Chang TC, Mendell JT, Salzberg SL. StringTie enables improved reconstruction of a transcriptome from RNA-seq reads. *Nat Biotechnol.* 2015;33(3):290-295.
