# Supplementary material for: Identification and validation of a prognostic signature of autophagy, apoptosis and pyroptosis-related genes for head and neck squamous cell carcinoma: to imply therapeutic choices of HPV negative patients
Source: Front Immunol. 2023 Jan 10;13:1100417. doi: 10.3389/fimmu.2022.1100417 (PMC9872116; doi:10.3389/fimmu.2022.1100417)
Supplement: Supplementary file 7 [file Table_1.docx]

**Table S1 Baseline clinic-pathological characteristics in TCGA dataset**

| **Characteristics** | **High CDI (N = 184)** | **Low CDI (N = 195)** | **Overall (N = 379)** | ***P*-value** |
| --- | --- | --- | --- | --- |
| **Age** |  |  |  | 0.695 |
| > 61Y | 85 (46.2%) | 94 (48.2%) | 179 (47.2%) |  |
| ≤ 61Y | 99 (53.8%) | 101 (51.8%) | 200 (52.8%) |  |
| **Gender** |  |  |  | 0.419 |
| Male | 130 (70.7%) | 145 (74.4%) | 275 (72.6%) |  |
| Female | 54 (29.3%) | 50 (25.6%) | 104 (27.4%) |  |
| **Smoking** |  |  |  | 0.319 |
| Yes | 103 (56.0%) | 119 (61.0%) | 222 (58.6%) |  |
| No | 81 (44.0%) | 76 (39.0%) | 157 (41.4%) |  |
| **Drinking** |  |  |  | 0.099 |
| Yes | 111 (60.3%) | 132 (67.7%) | 243 (64.1%) |  |
| No | 70 (38.0%) | 58 (29.7%) | 128 (33.8%) |  |
| NA | 3 (1.6%) | 5 (2.6%) | 8 (2.1%) |  |
| **Tumor site** |  |  |  | **0.014** |
| Oral cavity | 135 (73.4%) | 120 (61.5%) | 255 (67.3%) |  |
| Pharynx | 49 (26.6%) | 75 (38.5%) | 124 (32.7%) |  |
| **TNM stage** |  |  |  | **< 0.001** |
| III-IV | 159 (86.4%) | 138 (70.8%) | 297 (78.4%) |  |
| I-II | 25 (13.6%) | 57 (29.2%) | 82 (21.6%) |  |
| **Grade** |  |  |  | 0.785 |
| Low | 44 (23.9%) | 48 (24.6%) | 92 (24.3%) |  |
| High-intermediate | 138 (75.0%) | 141 (72.3%) | 279 (73.6%) |  |
| NA | 2 (1.1%) | 6 (3.1%) | 8 (2.1%) |  |
| **HPV status** |  |  |  | 0.847 |
| Positive | 27 (14.7%) | 30 (15.4%) | 57 (15.0%) |  |
| Negative | 157 (85.3%) | 165 (84.6%) | 322 (85.0%) |  |

‘NA’ represent the corresponding information is not available;

*P*-value is applied for the patients excepted for the ‘NA’.

**Table S2 Baseline clinic-pathological characteristics in GSE42743 dataset**

| **Characteristics** | **High CDI (N = 33)** | **Low CDI (N = 40)** | **Overall (N = 73)** | ***P*-value** |
| --- | --- | --- | --- | --- |
| **Age** |  |  |  | 0.766 |
| > 60Y | 16 (48.5%) | 18 (45.0%) | 34 (46.6%) |  |
| ≤ 60Y | 17 (51.5%) | 22 (55.0%) | 39 (53.4%) |  |
| **Gender** |  |  |  | 0.895 |
| Male | 26 (78.8%) | 31 (77.5%) | 57 (78.1%) |  |
| Female | 7 (21.2%) | 9 (22.5%) | 16 (21.9%) |  |
| **Smoking** |  |  |  | 0.898 |
| Yes | 26 (78.8%) | 32 (80.0%) | 58 (79.5%) |  |
| No | 7 (21.2%) | 8 (20.0%) | 15 (20.5%) |  |
| **Tumor site** |  |  |  | 0.673 |
| Oral cavity | 32 (97.0%) | 38 (95.0%) | 70 (95.9%) |  |
| Pharynx | 1 (3.0%) | 2 (5.0%) | 3 (4.1%) |  |
| **TNM stage** |  |  |  | 0.087 |
| III-IV | 28 (84.8%) | 27 (67.5%) | 55 (75.3%) |  |
| I-II | 5 (15.2%) | 13 (32.5%) | 18 (24.7%) |  |

**Table S3 Baseline clinic-pathological characteristics in GSE65858 dataset**

| **Characteristics** | **High CDI (N = 211)** | **Low CDI (N = 42)** | **Overall (N = 253)** | ***P*-value** |
| --- | --- | --- | --- | --- |
| **Age** |  |  |  | 0.414 |
| > 58Y | 101 (47.9%) | 23 (54.8%) | 124 (49.0%) |  |
| ≤ 58Y | 110 (52.1%) | 19 (45.2%) | 129 (51.0%) |  |
| **Gender** |  |  |  | 0.698 |
| Male | 176 (83.4%) | 34 (81.0%) | 210 (83.0%) |  |
| Female | 35 (16.6%) | 8 (19.0%) | 43 (17.0%) |  |
| **Smoking** |  |  |  | 0.450 |
| Yes | 176 (83.4%) | 33 (78.6%) | 209 (82.6%) |  |
| No | 35 (16.6%) | 9 (21.4%) | 44 (17.4%) |  |
| **Drinking** |  |  |  | 0.406 |
| Yes | 190 (90.0%) | 36 (85.7%) | 226 (89.3%) |  |
| No | 21 (10.0%) | 6 (14.3%) | 27 (10.7%) |  |
| **Tumor site** |  |  |  | 0.077 |
| Oral cavity | 70 (33.2%) | 8 (19.0%) | 78 (30.8%) |  |
| Pharynx | 139 (65.9%) | 33 (78.6%) | 172 (68.0%) |  |
| NA | 2 (0.9%) | 1 (2.4%) | 3 (1.2%) |  |
| **TNM stage** |  |  |  | 0.361 |
| III-IV | 168 (79.6%) | 36 (85.7%) | 204 (80.6%) |  |
| I-II | 43 (20.4%) | 6 (14.3%) | 49 (19.4%) |  |
| **HPV status** |  |  |  | **0.004** |
| Positive | 53 (25.1%) | 20 (47.6%) | 73 (28.9%) |  |
| Negative | 157 (74.4%) | 22 (52.4%) | 179 (70.8%) |  |
| NA | 1 (0.5%) | 0 (0%) | 1 (0.4%) |  |

‘NA’ represent the corresponding information is not available;

*P*-value is applied for the patients excepted for the ‘NA’.

**Table S4 Baseline clinic-pathological characteristics in dataset of Qilu Hospital of Shandong University**

| **Characteristics** | **High CDI (N = 21)** | **Low CDI (N = 7)** | **Overall (N = 28)** | ***P*-value** |
| --- | --- | --- | --- | --- |
| **Age** |  |  |  | 1.000 |
| > 61Y | 11 (52.4%) | 3 (42.9%) | 14 (50.0%) |  |
| ≤ 61Y | 10 (47.6%) | 4 (57.1%) | 14 (50.0%) |  |
| **Gender** |  |  |  | 0.418 |
| Male | 13 (61.9%) | 3 (42.9%) | 16 (57.1%) |  |
| Female | 8 (38.1%) | 4 (57.1%) | 12 (42.9%) |  |
| **Smoking** |  |  |  | 0.674 |
| Yes | 7 (33.3%) | 3 (42.9%) | 10 (35.7%) |  |
| No | 14 (66.7%) | 4 (57.1%) | 18 (64.3%) |  |
| **Drinking** |  |  |  | 1.000 |
| Yes | 6 (28.6%) | 2 (28.6%) | 8 (28.6%) |  |
| No | 15 (71.4%) | 5 (71.4%) | 20 (71.4%) |  |
| **Tumor site** |  |  |  | 0.545 |
| Oral cavity | 17 (81.0%) | 7 (100%) | 24 (85.7%) |  |
| Pharynx | 4 (19.0%) | 0 (0%) | 4 (14.3%) |  |
| **TNM stage** |  |  |  | 1.000 |
| III-IV | 5 (23.8%) | 1 (14.3%) | 6 (21.4%) |  |
| I-II | 16 (76.2%) | 6 (85.7%) | 22 (78.6%) |  |
| **Grade** |  |  |  | 0.288 |
| Low | 6 (28.6%) | 0 (0%) | 6 (21.4%) |  |
| High-intermediate | 15 (71.4%) | 7 (100%) | 22 (78.6%) |  |
